# Nasal Tissue Extraction Is Essential for Characterization of the Murine Upper Respiratory Tract Microbiota

**DOI:** 10.1128/mSphere.00562-20

**Published:** 2020-12-16

**Authors:** L. Patrick Schenck, Joshua J. C. McGrath, Daphnée Lamarche, Martin R. Stämpfli, Dawn M. E. Bowdish, Michael G. Surette

**Affiliations:** a Department of Biochemistry and Biomedical Sciences, McMaster University, Hamilton, Ontario, Canada; b Farncombe Family Digestive Health Research Institute, McMaster University, Hamilton, Ontario, Canada; c Michael G. Degroote Institute for Infectious Disease Research, McMaster University, Hamilton, Ontario, Canada; d McMaster Immunology Research Centre, McMaster University, Hamilton, Ontario, Canada; e Firestone Institute for Respiratory Health, McMaster University, Hamilton, Ontario, Canada; f Department of Pathology and Molecular Medicine, McMaster University, Hamilton, Ontario, Canada; g Department of Medicine, McMaster University, Hamilton, Ontario, Canada; University of Michigan—Ann Arbor

**Keywords:** *Streptococcus pneumoniae*, colonization, microbiota, upper respiratory tract

## Abstract

Respiratory infections are a leading cause of morbidity and mortality worldwide. Bacterial pathogens often colonize the upper respiratory tract (nose or mouth) prior to causing lower respiratory infections or invasive disease. Interactions within the upper respiratory tract between colonizing bacteria and the resident microbiota could contribute to colonization success and subsequent transmission. Human carriage studies have identified associations between pathogens such as Streptococcus pneumoniae and members of the resident microbiota, although few mechanisms of competition and cooperation have been identified and would be aided by the use of animal models. Little is known about the composition of the murine nasal microbiota; thus, we set out to improve assessment, including tissue sampling, composition, and comparison between mouse sources. Nasal washes were efficient in sampling the nasopharyngeal space but barely disrupted the nasal turbinates. Nasal tissue extraction increased the yield of cultivable bacterial compared to nasal washes, revealing distinct community compositions. Experimental pneumococcal colonization led to dominance by the colonizing pathogen in the nasopharynx and nasal turbinates, but the composition of the microbiota, and interactions with resident microbes, differed depending on the sampling method. Importantly, vendor source has a large impact on microbial composition. Bacterial interactions, including cooperation and colonization resistance, depend on the biogeography of the nose and should be considered during research design of experimental colonization with pathogens.

**IMPORTANCE** The nasal microbiota is composed of species that play a role in the colonization success of pathogens, including Streptococcus pneumoniae and Staphylococcus aureus. Murine models provide the ability to explore disease pathogenesis, but little is known about the natural murine nasal microbiota. This study established techniques to allow the exploration of the bacterial members of the nasal microbiota. The mouse nasal microbiota included traditional respiratory bacteria, including *Streptococcus*, *Staphylococcus*, and *Moraxella* species. Analyses were affected by different sampling methods as well as the commercial source of the mice, which should be included in future research design of infectious disease research.

## Abstract (precis)

The nasal microbiota is composed of species that play a role in the colonization success of pathogens, including Streptococcus pneumoniae and Staphylococcus aureus. Murine models provide the ability to explore disease pathogenesis, but little is known about the natural murine nasal microbiota.

## INTRODUCTION

The upper respiratory tract (URT) is the initial barrier against airway pathogens. Asymptomatic colonization by potential pathogens, including Streptococcus pneumoniae and Staphylococcus aureus, provides a reservoir capable of causing disease within the host or being transmitted to other carriers via respiratory droplets (1). Pathogens could inhibit colonization by other pathogens, as epidemiological studies have identified a negative correlation between S. pneumoniae and S. aureus in children (2, 3). Consistent with this, studies of pneumococcal vaccination have shown that the removal or reduction of S. pneumoniae is associated with increased S. aureus colonization (4). The contribution of the resident microbiota is relatively unknown, although a recent study demonstrated that a nasopharyngeal bacterial composition with high levels of *Corynebacterium* or *Dolosigranulum* species was correlated with a reduced risk of lower respiratory infections (5). The antipathogenic activities of *Corynebacterium* species have been identified *in vitro* (6, 7). A further understanding of the agonistic and antagonistic interactions between microbial species may explain why certain populations are more susceptible to colonization and infection.

Mechanistic studies involving pneumococcal colonization and the URT microbiota are difficult due to high interindividual variability, low bacterial biomass, and diverse topography. An experimental human pneumococcal colonization model identified increased α-diversity (higher microbial community diversity within a subject) in subjects with successful pneumococcal colonization (8). Identification of the contribution of individual bacterial species was challenging due to distinct microbial compositions between subjects. Mouse models allow for greater control over microbial composition and have been used to assess the alterations of the nasal microbiota during pneumococcal infection (9, 10); however, little is known about the naive URT microbiota composition in mice.

Assessment of murine nasal bacteria is predominated by nasal washes. Cannulation and flushing from trachea to nares, often called a nasal wash, are the most frequently used methods to assess nasal colonization, although tissue extraction is sometimes used to assess adherent or invasive pathogens (11–14). Differences in the duration of colonization and bacterial interactions have been identified using different tissue extraction methods (14–17). No studies have compared these two sampling methods for their efficiency at extracting the native microbiota and their impact on pathogen-microbiota associations. We used culture-dependent and -independent methods to assess the composition of the URT microbiota using nasal washes and complete tissue collection. Nasal washes were sufficient for sampling the nasopharynx but did not disrupt the turbinates. Nasal tissue collection yielded a distinct community with increased bacterial loads compared to those in nasal wash samples. We show that nasal wash may underestimate the presence and interactions of Streptococcus pneumoniae within the URT. Furthermore, the URT microbiota composition differs by the source of the mice. This approach to microbial analysis of the murine URT will improve the study design of host-pathogen and pathogen-commensal interactions during colonization.

## RESULTS

### Nasal wash does not completely disrupt nasal surfaces.

**FIG 1:**
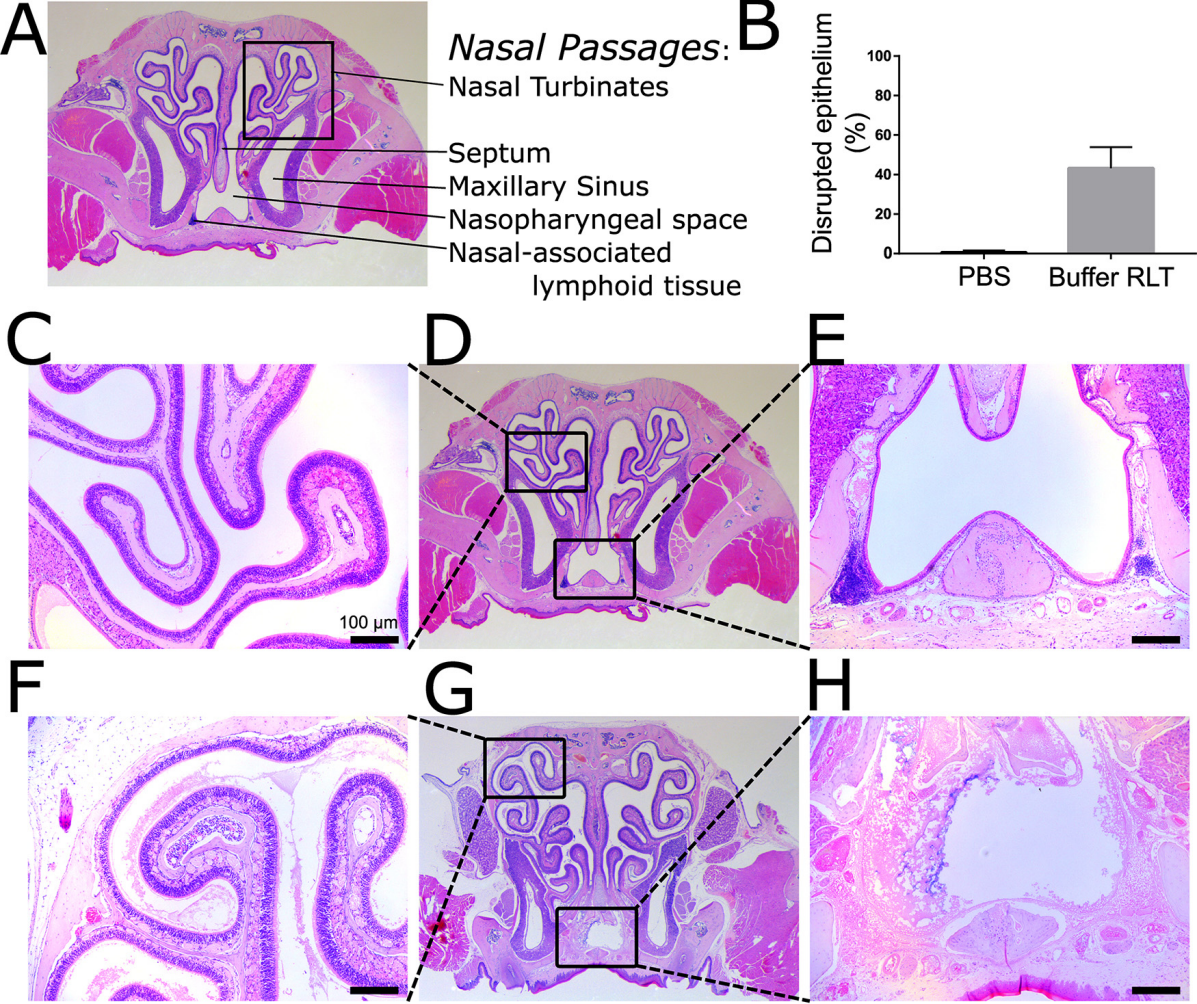
Nasal wash does not effectively sample the nasal cavity. (A) Nasal cavities were washed with PBS (*n* = 3) and buffer RLT (*n* = 3), and tissues were collected for histological analysis. (B) Hematoxylin-and-eosin-stained cross sections demonstrate that nasal washes with PBS do not disrupt the epithelial layer compared to buffer RLT. (C to E) Specifically, PBS washes do not impact the epithelial architecture in the turbinates (C and D) or the nasopharyngeal space (E). (F to H) Buffer RLT washes greatly disrupt the nasopharyngeal space (G and H) but not the nasal turbinates (F).

The biogeography affected by a nasal wash is unknown. We performed nasal washes using a gentle buffer (phosphate-buffered saline [PBS]) or a harsh buffer (buffer RLT), followed by histological assessment of the nasal tissue via hematoxylin-and-eosin staining (Fig. 1A and B). PBS does not affect the epithelial architecture of the nasal cavity (Fig. 1C to E), whereas buffer RLT disrupts the epithelial layer within the nasopharyngeal space and septum as well as the nasal-associated lymphoid tissue (Fig. 1G and H) but not the nasal turbinates (Fig. 1F). Overall, nasal washes with harsh buffers were unable to disrupt a large majority of the epithelial layer in the nasal cavity, implying that nasal washes do not accurately sample the complete biogeography of the nasal tissue (Fig. 1A and B).

### Nasal tissue extraction recovers more bacteria than nasal washes.

**FIG 2:**
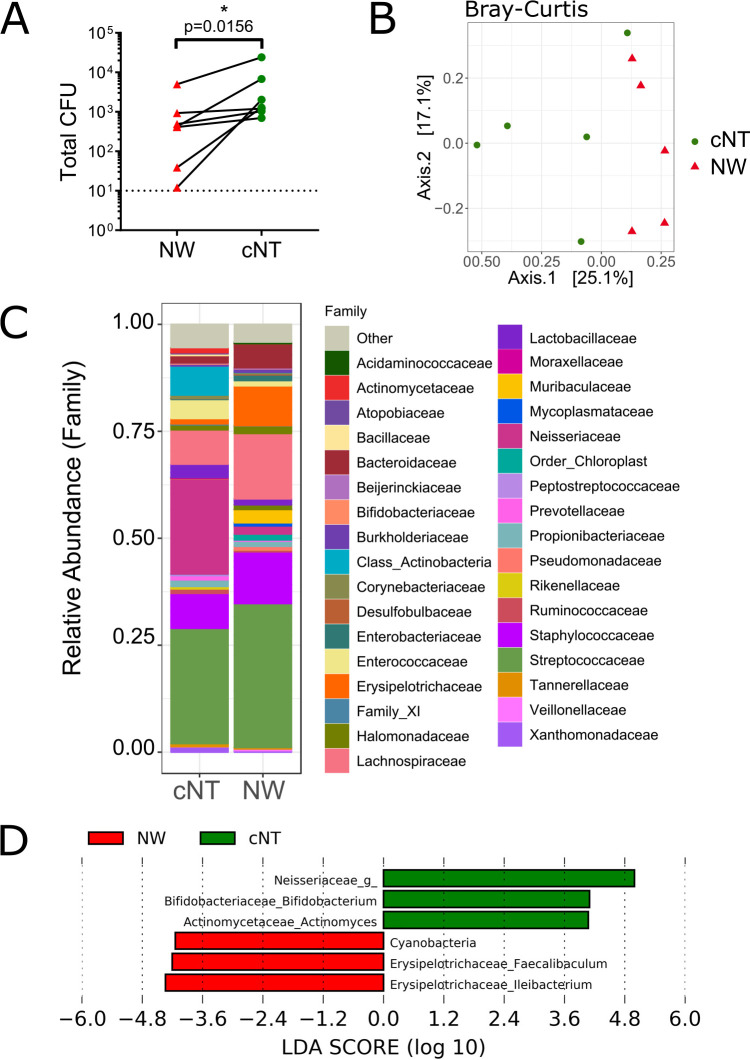
Nasal tissue extraction is essential for complete microbiota assessment. Nasal wash samples were collected using PBS, followed by tissue collection from the same mice and V3 16S rRNA gene high-throughput sequencing. (A) Nasal tissue had significantly more bacteria than nasal wash (NW) samples. (B) Nasal tissue microbial communities clustered separately from nasal wash communities (*P* < 0.05 by Bray-Curtis PERMANOVA). (C and D) Nasal tissue microbiota were enriched in *Neisseriaceae*, *Bifidobacterium*, and *Actinomyces*, while nasal wash samples were enriched in *Erysipelotrichaceae* and *Cyanobacteria* (LEfSe).

Nasal wash and nasal tissue samples were homogenized, plated on brain heart infusion (BHI) agar and fastidious anaerobic agar (FAA), and incubated aerobically and anaerobically, respectively. Overall, the bacterial load was low in both complete nasal tissue (cNT) and nasal wash samples (∼10^3^ CFU/mouse). Nasal tissue had significantly higher bacterial loads and diversity of colony morphotypes than nasal wash samples (Fig. 2A). Complementary to culture-based analysis, 16S rRNA gene sequencing revealed that cNT and nasal wash samples clustered separately from each other (Fig. 2B) (*P* < 0.05 by permutational multivariate analysis of variance [PERMANOVA]; *R*^2^ = 0.181). *Streptococcus* species and *Staphylococcus* species were dominant in nasal tissue and wash samples (Fig. 2C). This difference in microbial composition was driven by nasal tissue containing significantly more *Neisseriaceae*, *Actinomyces*, and *Bifidobacterium* species, while nasal wash samples contained more *Erysipelotrichaceae* and *Cyanobacteria* (Fig. 2D) (linear discriminate analysis [LDA] effect size [LEfSe]). No difference was seen in Shannon diversity (*P* = 0.26) or observed species (*P* = 0.4181) between nasal wash and nasal tissue samples (see Fig. S1 in the supplemental material).

10.1128/mSphere.00562-20.1 FIG S1 No difference in α-diversity between nasal tissue and nasal wash microbiota. Nasal wash samples were collected using PBS, followed by tissue collection from the same mice and V3 16S rRNA gene high-throughput sequencing. There was no difference in observed species (*P* = 0.4181) or Shannon diversity (*P* = 0.26). Each dot represents one mouse. Data were analyzed by Mann-Whitney tests. Download FIG S1, TIF file, 0.04 MB. Copyright © 2020 Schenck et al. 2020 Schenck et al. This content is distributed under the terms of the Creative Commons Attribution 4.0 International license.

### The mouse nasal microbiota is distinct from the gut microbiota.

It has been reported that a significant amount of the bacterial DNA found in the URT is from environmental contamination rather than being due to resident microbes (18). In mice, contaminating DNA can come from exposure to fecal matter and/or reagent controls. To determine the degree to which microbial profiles are influenced by fecal exposure, we compared the nasal tissue and gut microbiota within a mouse. The nasal microbiota was distinct from the gut microbiota (Fig. S2A) (*P* < 0.001 by PERMANOVA; *R*^2^ = 0.383). The gut microbiota has greatly increased α-diversity compared to the nasal microbiota (Fig. S2B). Additionally, the dominant families in the gut (*Muribaculaceae*, *Lactobacillaceae*, *Lachnospiraceae*, and *Erysipelotrichaceae*) are different from the dominant families in the nasal tissue (*Streptococcaceae*, *Staphylococcaceae*, and *Enterococcaceae*) microbiota (Fig. S2C).

10.1128/mSphere.00562-20.2 FIG S2 The murine nasal microbiota is distinct from the gut microbiota. DNA was extracted from nasal and cecal tissues (*n* = 9 mice), and the V3 region of the 16S rRNA gene was sequenced. (A) The microbial communities were distinct between the nasal and cecal microbiota (*P* < 0.001 by PERMANOVA). (B) The alpha-diversity metrics demonstrate greatly increased richness in the cecal microbiota compared to the nasal microbiota (*P* < 0.001 by a Mann-Whitney test). (C) Taxon summary comparing the family-level identifications of cecal and nasal microbiota. Download FIG S2, EPS file, 0.2 MB. Copyright © 2020 Schenck et al. 2020 Schenck et al. This content is distributed under the terms of the Creative Commons Attribution 4.0 International license.

### The mouse nasal microbiota is distinct from extraction and sequencing controls.

Sequencing and extraction controls are essential for low-biomass microbiota analysis as reagents and tissue handling have been shown to influence the community composition of low-biomass communities (19, 20). In this study, negative extraction (surgical tools dipped in PBS and then exposed to the same extraction process as tissues, cage bedding, and drinking water) and PCR negative (elution water used instead of the DNA template) samples were included to determine the impact of contaminating DNA. The negative samples were distinct from the nasal tissue microbiota (Fig. S3A) (*P* < 0.05 by PERMANOVA; *R*^2^ = 0.138). An unweighted pair group method with arithmetic mean (UPGMA) tree based on Bray-Curtis distances demonstrated the separation between negative controls and samples (Fig. S3B). The dominant taxa in the nasal tissue, namely, *Streptococcus* and *Staphylococcus*, are not present in the negative samples (Fig. S3C). The inclusion of negative samples with every extraction is still worthwhile to distinguish low-abundance communities from reagent contamination.

10.1128/mSphere.00562-20.3 FIG S3 Sequencing and extraction negative controls are distinct from the nasal microbiota. DNA was extracted from nasal tissues, bedding material, drinking water, PBS-exposed surgical tools, or water used in place of the template prior to amplification and sequencing of the V3 region of the 16S rRNA gene. (A) The communities were distinct via Bray-Curtis principal-component analysis (PCoA) (*P* < 0.05 by PERMANOVA; *R*^2^ = 0.138). (B) UPGMA tree based on Bray-Curtis distances demonstrating that the negative samples are distinct from the nasal tissue microbiota. (C) Taxonomic summaries of the negative samples contain several different taxa compared to the nasal tissue. Download FIG S3, EPS file, 0.2 MB. Copyright © 2020 Schenck et al. 2020 Schenck et al. This content is distributed under the terms of the Creative Commons Attribution 4.0 International license.

### Mouse source affects the composition of the nasal microbiota.

**FIG 3:**
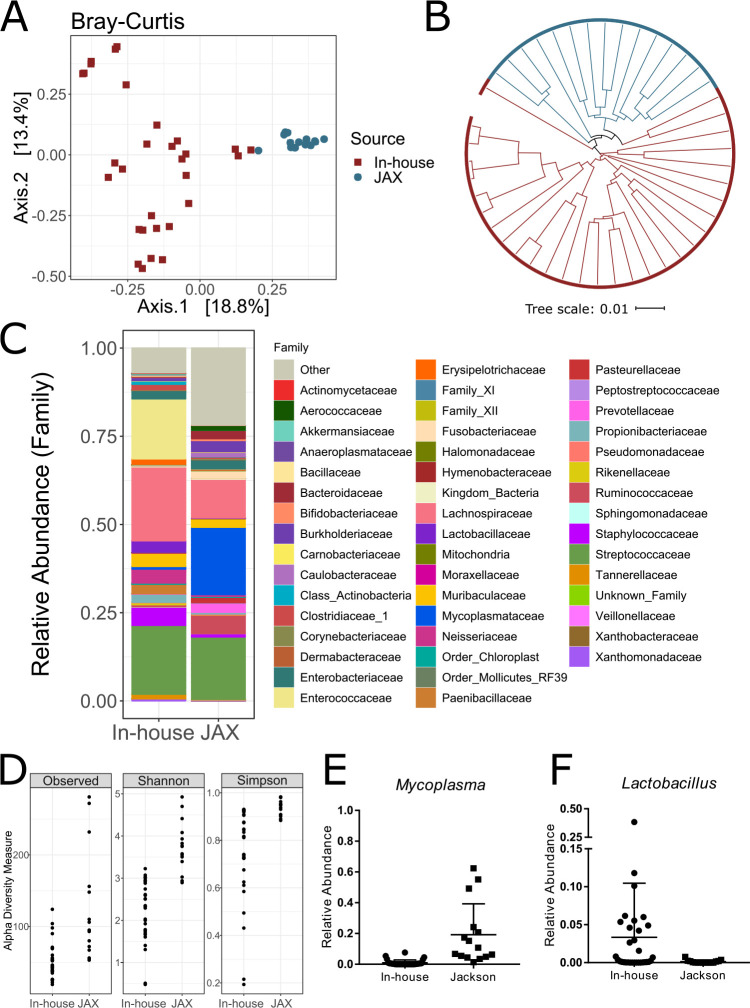
Source of mice impacts nasal microbiota composition. The nasal microbiota of C57BL/6J mice ordered directly from Jackson Laboratories were compared to those of C57BL/6J mice bred in-house for several generations. (A) JAX mice had a significantly distinct microbiota composition (*P* < 0.0001 by PERMANOVA). (B) Bray-Curtis distance tree demonstrating the clustering of mice from Jackson Laboratories compared to mice bred in-house. (C) Taxon summary at the family level comparing the nasal microbiota of in-house-bred mice to those of JAX mice. (D) Alpha-diversity is increased in Jackson Laboratories murine nasal microbiota compared to those of in-house-bred mice, as measured by observed species, Chao1, and Shannon diversity (*P* < 0.001 by a Mann-Whitney test). LEfSe analysis revealed 109 genera within the nasal microbiota that were significantly different between in-house mice and Jackson Laboratories mice. (E and F) The nasal microbiota of mice from Jackson Laboratories were enriched in *Mycoplasma* (E) and decreased in *Lactobacillus* (F) species compared to mice bred in-house.

**FIG 4:**
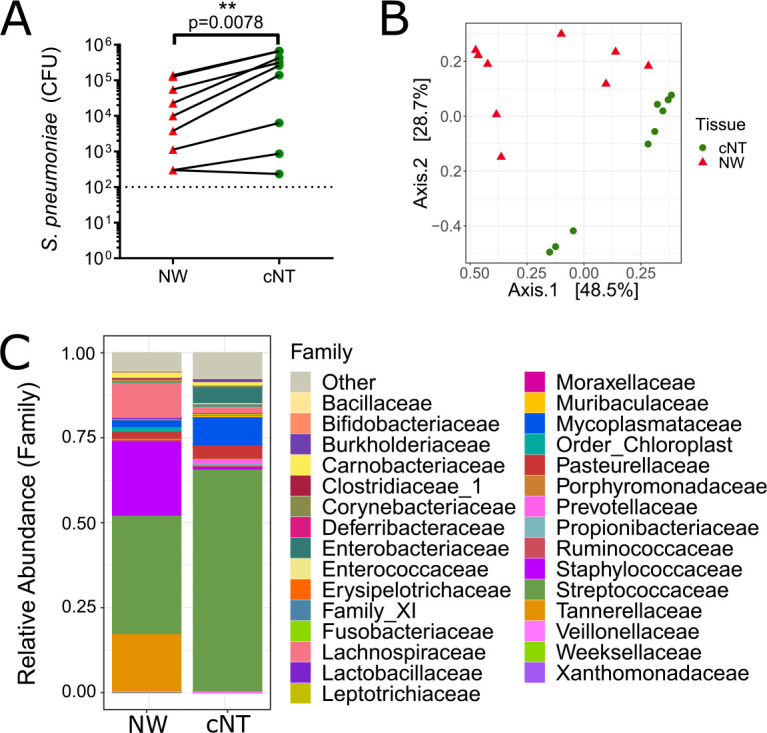
Streptococcus pneumoniae dominates the nasal microbiota during colonization. Female C57BL/6J mice were intranasally colonized with 10^7^ CFU of S. pneumoniae and sacrificed 3 days later for collection of PBS nasal wash samples and complete nasal tissues (*n* = 9). (A) Nasal tissue had significantly higher pneumococcal loads than the nasal wash samples (*P* = 0.0078 by a Wilcoxon match-paired signed-rank test). (B) The nasal tissue microbiota was distinct from the nasal wash microbiota (*P* = 0.0002 by PERMANOVA). (C) Summary taxon plot of the nasal tissue microbiota community (dominated by *Streptococcaceae* and *Mycoplasmataceae*) and the PBS nasal wash microbiota (dominated by *Tannerellaceae*, *Streptococcaceae*, and *Staphylococcaceae*).

Differences in the murine gut microbiota between breeding sites have been identified as a source of experimental variability (21). We compared the nasal tissue microbiota from mice bred at McMaster University (*n* = 32) to those of mice ordered from Jackson Laboratories (JAX) (*n* = 15). JAX mice have a significantly different nasal microbiota composition compared to in-house-bred mice (Fig. 3A and B) (*P* < 0.0001 by PERMANOVA; *R*^2^ = 0.226). While both groups of mice are dominated by *Streptococcaceae*, JAX mice have several other dominant taxa (Fig. 3C). Furthermore, JAX mice had increased α-diversity within the nasal tissue microbiota (*P* < 0.001 by a Mann-Whitney test) (Fig. 4D). LEfSe analysis revealed that 109 genera were significantly different between JAX and in-house-bred mice, including *Mycoplasma* (Fig. 4E) and *Lactobacillus* (Fig. 4F).

### Streptococcus pneumoniae colonization leads to domination of the nasal microbiota.

Streptococcus pneumoniae colonizes the upper respiratory tract, a process which disrupts preexisting microbial communities prior to causing respiratory and invasive infections (9, 10). Investigation into microbial interactions between S. pneumoniae and other nasal microbiota members has been primarily performed by analyzing nasal wash samples, which may overlook microbe-microbe interactions occurring deeper in the nasal tissue. Mice were intranasally colonized with Streptococcus pneumoniae, and nasal wash and tissue samples were collected after 3 days (*n* = 9 mice). The nasal tissue had higher levels of S. pneumoniae than the paired nasal wash sample (Fig. 4A). The cNT and nasal wash microbiota of colonized mice were distinct (Fig. 3B). S. pneumoniae dominated the nasal tissue (Fig. 4B and C). LEfSe analysis revealed 24 significantly different genera between the two sampling methods (Fig. S4). Spearman correlations identified that the relative abundance of *Streptococcus* sequence reads strongly correlated with cultured S. pneumoniae in the cNT (*R* = 0.9) and nasal wash (*R* = 0.95) samples (Fig. S5). Furthermore, nasal tissue S. pneumoniae CFU correlated with nasal wash CFU (*R* = 0.95), and *Streptococcus* amplicon sequence variant (ASV) relative abundances were correlated between nasal tissue and wash samples (*R* = 0.88).

10.1128/mSphere.00562-20.4 FIG S4 LEfSe results of PBS nasal wash and tissue microbiota differences in S. pneumoniae-colonized mice. Download FIG S4, EPS file, 0.3 MB. Copyright © 2020 Schenck et al. 2020 Schenck et al. This content is distributed under the terms of the Creative Commons Attribution 4.0 International license.

10.1128/mSphere.00562-20.5 FIG S5 Strong Spearman correlations between cultured S. pneumoniae and *Streptococcus* ASV relative abundances. Mice were colonized with S. pneumoniae, and PBS nasal wash samples and tissues were collected 3 days later. (A and B) S. pneumoniae in the nasal tissue strongly correlated with *Streptococcus* ASV relative abundances in the nasal tissue (A) and nasal wash (B) samples. (C) The relative abundances of *Streptococcus* ASVs are strongly correlated between the nasal wash and nasal tissue samples. (D) Cultured S. pneumoniae CFU in the nasal tissue are strongly correlated with cultured S. pneumoniae CFU in the nasal wash samples. Each dot represents one mouse (*n* = 9). Download FIG S5, EPS file, 0.3 MB. Copyright © 2020 Schenck et al. 2020 Schenck et al. This content is distributed under the terms of the Creative Commons Attribution 4.0 International license.

### Nasal microbiota correlations are dependent on the tissue extraction methodology.

**FIG 5:**
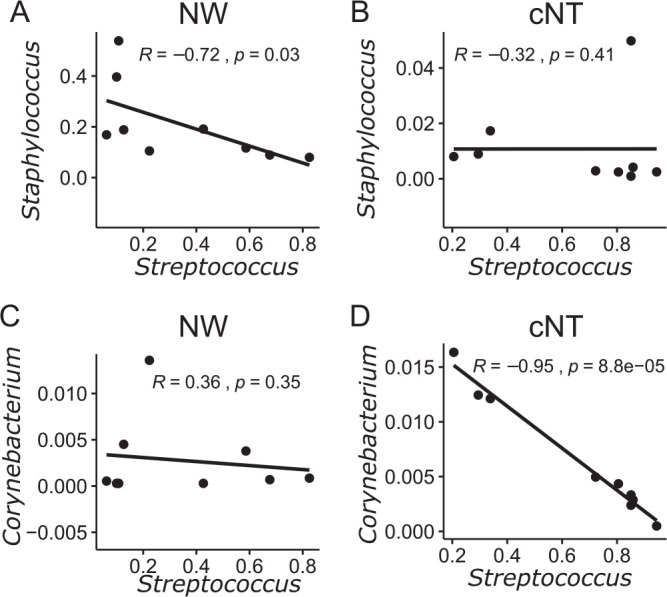
Microbiota interactions depend on the nasal sampling method. Spearman correlations of relative abundances of ASVs in nasal wash samples and nasal tissues of S. pneumoniae*-*colonized mice were determined. (A and B) *Staphylococcus* and *Streptococcus* species have a negative correlation in the PBS nasal wash samples (A) but not nasal tissue (B). (C and D) *Corynebacterium* species have a negative correlation in the nasal tissue (D) but not the nasal wash samples (C). Each dot represents the relative abundance within one animal, from either nasal wash or tissue samples (*n* = 9).

*Staphylococcus* species decrease during Streptococcus pneumoniae colonization when sampled by nasal wash (9, 10). *Staphylococcus* and *Streptococcus* species were negatively correlated in the nasal wash samples of S. pneumoniae-colonized mice (*R* = −0.72; *P* = 0.03) (Fig. 5A); however, there was no significant correlation for the nasal tissue microbiota (*R* = −0.32; *P* = 0.41) (Fig. 5B). *Corynebacterium* species are overrepresented in the nasal microbiota of children and adults negative for pneumococcal colonization and have recently been identified to inhibit the growth of S. pneumoniae *in vitro* (7). No correlation was found between *Corynebacterium* and *Streptococcus* species in the nasal wash microbiota of pneumococcus-colonized mice (*R* = 0.36; *P* = 0.35) (Fig. 5C); however, a strong negative correlation (*R* = −0.95; *P* = 8.8 × 10^−5^) between *Corynebacterium* species and *Streptococcus* species existed in the nasal tissue (Fig. 5D), suggesting that interactions between these species could occur within regions not affected by nasal washes. These correlations imply that both sampling techniques may be necessary to uncover microbial interactions that are site specific. Furthermore, niche-specific interactions may be driving antagonism or cooperation between S. pneumoniae and other bacterial species in the URT.

## DISCUSSION

The upper respiratory tract plays an essential role in breathing, trapping inhaled microbes and particles while heating and humidifying air prior to entering the lungs. The topography provides different niches for bacteria to colonize, resulting in protective or deleterious interactions with other microbial species. This study identified that nasal washes do not completely sample the murine nasal microbiota and may overlook some interactions occurring within the turbinates or submucosa of the nasal tissue. Differences in humidity, mucus secretion, and epithelial cell type could contribute to the colonization success of various pathogens and commensals (22, 23). In humans, sampling of different regions of the nasal cavity distinguished the microbial composition of the anterior nares from that of the middle meatus and sphenoethmoidal recess (24). The middle meatus and sphenoethmoidal recess are dominated by *Corynebacterium* and *Staphylococcus* species, while anterior nares have a greater abundance of *Propionibacterium*/*Cutibacterium* species. In this study, *Actinomyces* and *Neisseria* species were increased in the nasal tissue, whereas the nasal wash samples had greater abundances of *Erysipelotrichaceae* family members. *Actinomyces* and *Neisseria* species in the human nasal microbiota have been associated with an increased risk of acute otitis media.

Select bacterial groups, including *Corynebacterium*, *Staphylococcus*, *Streptococcus*, *Dolosigranulum*, and *Moraxella* species, are commonly found in the human nose (25, 26). The murine nasal microbiota has a dominant *Staphylococcus* and *Streptococcus* population but small *Corynebacterium*, *Dolosigranulum*, and *Moraxella* species populations, similar to mice obtained from Jackson Laboratories and other facilities (9). Mice can be experimentally colonized by *Corynebacterium* and *Moraxella* species, indicating that the low abundance in specific-pathogen-free (SPF) mice could be due to a lack of exposure (27, 28). A major determinant of colonization is adherence, requiring selective interactions between host and bacterial factors. Neisseria meningitidis and Streptococcus pyogenes colonization in mice requires the expression of human-specific adherence factors in the olfactory epithelium (12, 29).

The composition of the microbiota differed between mice bred in-house and those delivered by Jackson Laboratories. Vendor-specific microbiota differences have been identified in gut and lung studies and have been implicated in altering the outcomes of disease models (18, 30, 31); however, our study is the first to demonstrate vendor-specific microbiome differences within the nasal tissue. Housing under SPF conditions at different facilities restricts access to many bacteria beyond pathogens, and SPF mice have drastically different respiratory microbiota compositions compared to wild mice (32). Indeed, mice captured from the wild have more, as well as different, bacteria in their lungs, which influences alveolar structure (32). The impact of differential colonization between facilities and sources may impact the baseline physiology or expression of some genes. Krone et al. previously assessed the murine nasal microbiota by nasal wash and found patterns similar to those in our mice, with high levels of *Staphylococcaceae*, *Streptococcaceae*, and *Erysipelotrichaceae*, but failed to detect *Actinomycetaceae* (9), which were detected in the nasal tissue samples in this study. Interestingly, Weyrich et al. found high levels of *Actinomycetaceae* (as well as *Streptococcaceae* and *Staphylococcaceae*) in nasal tissue samples from their facility (33). Whether these differences are due to different breeding facilities, housing conditions, or sampling techniques is unclear.

Sequencing-based analysis of the URT microbiota is complicated due to the low microbial biomass compared to host DNA (34). Extraction and amplification methods will affect the outcome of sequencing results, and the inclusion of template negative controls is essential for determination of the microbial composition. We demonstrated that the nasal tissue microbiota is distinct from the gut microbiota and negative controls. Furthermore, there was a strong correlation between the cultivable amount of inoculated S. pneumoniae and the ability to detect *Streptococcus* ASVs in the nasal tissue and wash samples. This strongly suggests that culture-independent analysis of the nasal tissue microbiota is representative of the cultivable microbiota and not contaminating DNA sequences from environmental or extraction sources.

Colonization is essential during bacterial pathogenesis, including adherence prior to invasion or spread to new hosts. Capsule expression by Streptococcus pneumoniae varies depending on nasal location, which alters its ability to adhere, evade killing, or be transmitted to a new host (35–38). As such, the detection of S. pneumoniae in nasal wash or tissue extraction samples has different implications for transmission versus invasive disease. Multiple studies have reported a negative correlation between *Staphylococcus* and *Streptococcus* species, potentially due to hydrogen peroxide production (39, 40). We have shown that this negative correlation exists in the nasopharynx of colonized mice but not in the nasal tissue. Conversely, we have shown a negative correlation between *Streptococcus* and *Corynebacterium* species in the nasal tissue but not in the nasal wash samples. *Corynebacterium* species have been demonstrated to liberate host triacylglycerols that kill S. pneumoniae (7). Previous studies have also implicated differences in Haemophilus influenzae and S. pneumoniae antagonism depending on the sampling method (15, 16). Together, these data suggest that multiple sampling methods may be necessary to determine bacterial interactions within the URT. Human experimental colonization models assess pneumococcal colonization, as well as microbial communities, via nasal wash (41) and identify many antagonistic interactions that have also been seen in murine models of experimental colonization (e.g., negative correlation of *Corynebacterium* and *Streptococcus* [41, 42]); however, whether there are microbial interactions missed by this sampling method is unclear. The structure of murine nasal tissue is much more complicated than that of human nasal tissue, so species-specific sampling techniques may be needed to study microbial interactions.

Overall, mechanistic investigation of the URT microbiota in infection and immunity requires animal models. Current studies have used mixed methodologies, including different sampling techniques and mouse vendors, to investigate development and disease phenotypes. Proper extraction and assessment of the nasal tissue of mice are essential to reveal reproducible, mechanistic interactions between host cells and microbial members. Our findings demonstrate that assessment of the nasal microbiota is dependent on biogeography and needs to be integrated into research design for evaluation of pathogen and commensal colonization and interactions.

## MATERIALS AND METHODS

### Animals.

C57Bl/6J mice were bred within the McMaster Central Animal Facility, except for the experiments in which we used female C57Bl/6J mice from Jackson Laboratories (Bar Harbor, ME). Mice from Jackson Labs were 6 to 8 weeks old and acclimated to specific pathogen-free conditions for 2 weeks prior to experiments. All mice had access to food and water *ad libitum*. All mice used in this study were female mice aged 8 to 12 weeks. Mice were anesthetized using isoflurane and euthanized by exsanguination. All experiments were approved by McMaster University’s Animal Research Ethics Board according to the recommendations of the Canadian Council for Animal Care.

### Tissue collection.

Nasal wash was completed as previously described (43). Briefly, a PE-20 polyethylene tube attached to a 26-gauge needle was inserted through a small incision in the trachea. Lavages were performed with 300 μl of sterilized phosphate-buffered saline (PBS) or buffer RLT (Qiagen), which was flushed through the trachea and collected through the nares in a 1.7-ml microtube. Complete nasal tissue (cNT) was collected from lavaged or naive mice via bisection of the skull with sterilized surgical tools (44). Excised tissues were homogenized in 300 μl PBS in 2-ml screw-top tubes using 2.8-mm ceramic beads for 1 min at 2,000 rpm (MoBio).

### Histological analysis.

After nasal washes, mouse heads were placed into formalin for 24 h before being placed in a Shandon TBD-2 decalcifier (Thermo Scientific, Kalamazoo, MI) for 4 days. Decalcified heads were placed in formalin for 2 days, followed by twice-daily washes with PBS for an additional 4 days. Samples were washed with and placed in 70% ethanol prior to standard histological processing and embedded in paraffin wax. Cross-sectional slices (5 μm) were mounted on slides and stained with hematoxylin and eosin according to standard protocols. Slides were randomized and scored in a blind fashion. The integrity of the epithelial lining was measured by quantifying the area of intact/disrupted epithelium using ImageJ.

### DNA extraction, amplification, and analysis of the 16S rRNA gene.

DNA was extracted from PBS nasal wash and cNT homogenates as previously described (10). Samples were mechanically homogenized with 0.2 g of 0.1-mm glass beads and 0.2 g of 2.8-mm glass beads in 800 μl of 200 mM NaPO_4_ (pH 8) and 100 μl of guanidine thiocyanate-EDTA-*N-*lauroyl sarcosine. After homogenization, the sample was incubated with 50 μl of lysozyme (100 mg/ml) and 10 μl of RNase A (10 mg/ml) for 1 h at 37°C, followed by incubation with a solution containing 25 μl sodium dodecyl sulfate (25%), 62.5 μl NaCl (5 M), and 25 μl proteinase K (20 mg/ml) for 1 h at 65°C. Samples were centrifuged for 5 min at maximum speed, and the supernatant was transferred to 900 μl of buffered phenol-chloroform-isoamyl alcohol (25:24:1). Samples were vortexed and centrifuged for 10 min at maximum speed prior to transferring the aqueous phase to DNA Clean and Concentrator-25 columns (Zymo) according to the manufacturer’s instructions, except that samples were eluted with 50 μl ultrapure water. Negative controls included PBS-exposed surgical tools, cage bedding, drinking water, and no-template PCR amplicons.

PCR amplification of the 16S rRNA gene (V3 region) involved a two-step, nested PCR, which improves efficiency in the presence of high host DNA levels (45). The first step involved the amplification of the 16S rRNA gene region spanning V1 to V5 using universal primers 8F (AGAGTTTGATCCTGGCTCAG) and 926R (CCGTCAATTCCTTTRAGTTT) for 15 cycles (94°C for 30 s, 56°C for 30 s, and 72°C for 60 s). This product was used as the template in the second reaction for 25 cycles (94°C for 30 s, 47°C for 30 s, and 72°C for 40 s) to amplify the V3 region of the 16S rRNA gene in preparation for MiSeq (Illumina) sequencing. Barcoded primer sequences were adapted similarly to previous work (46). All reactions, including extraction negative and no-template controls, were performed in triplicate to reduce PCR bias, and reaction mixtures were pooled prior to sequencing using the MiSeq sequencing platform (Illumina, Inc., San Diego, CA) at the Farncombe Genomics Facility at McMaster University. Sequences were trimmed using CutAdapt (47) prior to analysis with DADA2 (48) to organize sequences into amplicon sequence variants (ASVs). Taxonomy was assigned using the Silva database (49). Relative abundance, α- and β-diversity, and rarefactions were completed using the Phyloseq package (50) in R version 3.5.2 (51). A dendrogram was constructed based on unweighted pair group method with arithmetic mean (UPGMA) hierarchical clustering using Bray-Curtis distances using the phangorn package (52) and plotted with iTOL (53). Differences between bacterial communities were tested using permutational multivariate analysis of variance (PERMANOVA) within the vegan package (54). Differences in taxon abundances were assessed using linear discriminate analysis (LDA) effect size (LEfSe) (55).

### Streptococcus pneumoniae colonization.

Mice were intranasally colonized with S. pneumoniae strain P1547 (serotype 6A), obtained from Jeff Weiser (NYU School of Medicine), as previously described (43). S. pneumoniae was grown in tryptic soy broth in a 5% CO_2_ incubator at 37°C until cultures were in late log phase (optical density at 600 nm [OD_600_] = 0.5). Cultures were spun down at 15,000 × *g* for 1 min and resuspended at a concentration of 10^9^ CFU/ml in PBS. Mice were colonized by depositing 10 μl containing 10^7^ CFU of S. pneumoniae directly in the nares. Mice were sacrificed at 3 days postinoculation prior to PBS nasal wash and cNT sample collection.

### Bacterial culture.

PBS nasal wash and tissue homogenates were incubated overnight on brain heart infusion agar or tryptic soy agar supplemented with 5% sheep’s blood and neomycin (10 μg/ml) or on prereduced fastidious anaerobic agar in an anaerobic chamber. Colonies were counted and collected via the addition of 1 ml BHI medium and scraping the plate surface with a cell scraper. A portion of the collected colonies was frozen at −80°C in 10% glycerol, while the remainder was prepared for genomic extraction.

### Statistical analysis.

GraphPad Prism 6 and R were used for statistical analysis. Nonparametric tests were used for comparison of histology scoring and bacterial plate counts. Differences with *P* values of <0.05 were considered statistically significant. Microbial community composition differences were determined using the Adonis function (PERMANOVA) with 10,000 permutations.

### Data availability.

Data related to this study have been deposited in the NCBI database under BioProject accession number PRJNA679949.

10.1128/mSphere.00562-20.6 FIG S6 Individual taxon plots of nasal tissue and PBS nasal wash samples from S. pneumoniae-colonized mice. Download FIG S6, EPS file, 0.2 MB. Copyright © 2020 Schenck et al. 2020 Schenck et al. This content is distributed under the terms of the Creative Commons Attribution 4.0 International license.
