# Supplementary figures and images for: Nasal Tissue Extraction Is Essential for Characterization of the Murine Upper Respiratory Tract Microbiota
Source: mSphere. 2020 Dec 16;5(6):e00562-20. doi: 10.1128/mSphere.00562-20 (PMC7771231; doi:10.1128/mSphere.00562-20)

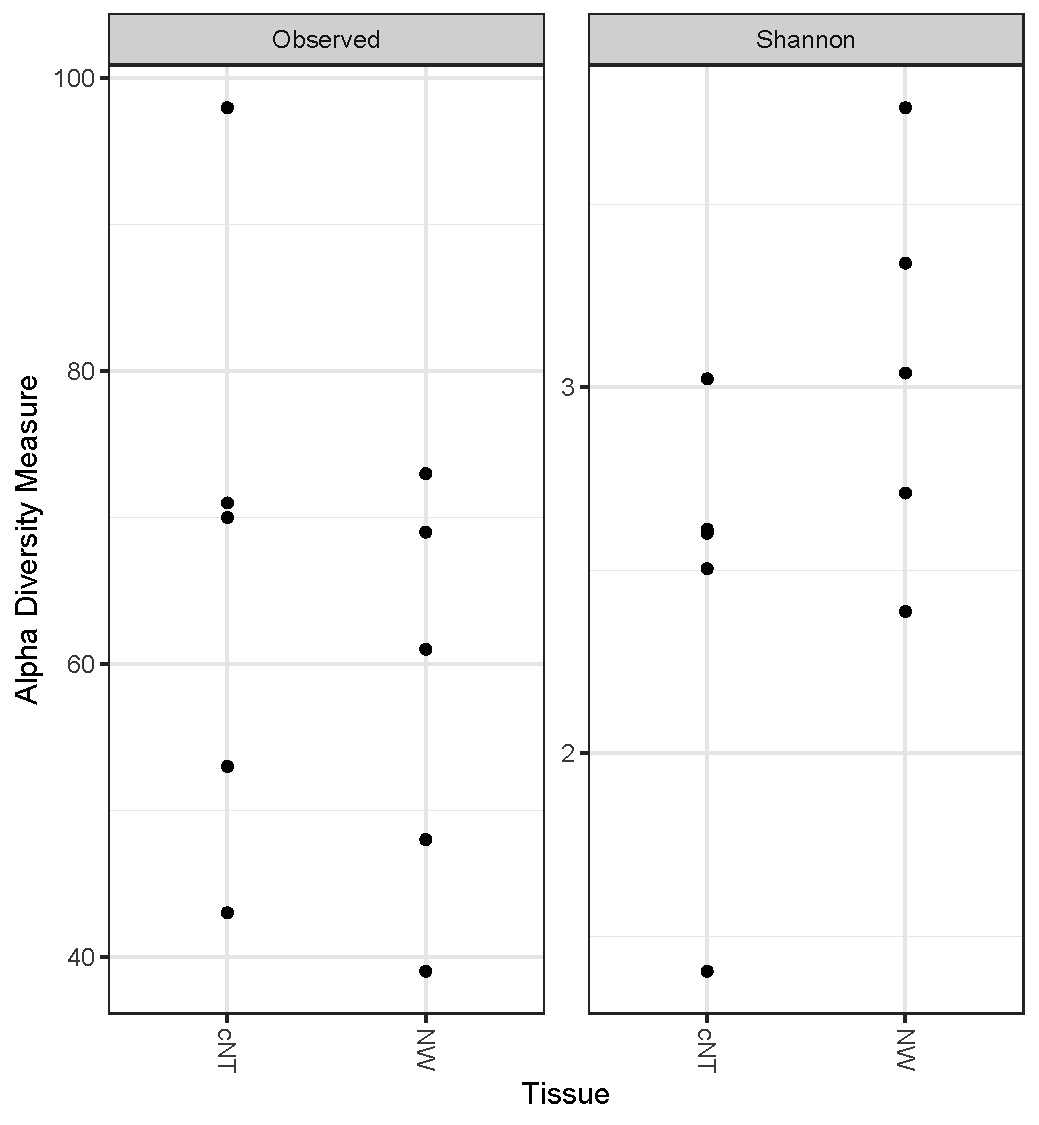

Supplement: FIG S1 [file mSphere.00562-20-sf001.tif]
